# Electric Light Good for the Eyes

**Published:** 1881-02

**Authors:** 


### ELECTRIC LIGHT GOOD FOR THE EYES.

When the electric light first began to be used in our shops, factories, and places of amusement, it was confidently asserted by its opponents that so dazzling a light must be injurious to the eye. The objection seemed plausible at least, although the light when diffused seemed to have the quality of bright moonlight, which is the reverse of irritating. People would persist in looking at the source of the light, and as the early lamps were far from steady the observer's eyes suffered both from the intensity of the light and the sudden and large variations in the quantity of it. It appears, however, from the experiments recently made by Professor Cohn, of Breslau, whose name is so familiar in connection with the investigation of color blindness and other optical defects, that our eyes will be benefitted rather than hurt by the new method of lighting, and it is obvious that with incandescent electric lighting the advantages will be still more marked.

While testing the influence of electric light on visual perception and the sense of color, Dr. Cohn proved, he thinks, that letters, spots, and colors were perceived at a much greater distance under electric illumination than by gas light, or even daylight. Compared with daylight, the electric light increased the sensation of yellow sixtyfold, red sixfold, and green and blue about twofold. Eyes that in daylight or gaslight could perceive and distinguish colors only with difficulty were much aided by the electric light, and the visual perception was much strengthened. In all cases of distant signaling, Dr. Cohn believes that the electric light will prove exceedingly and especially useful.—*Scientific American.*

